# Exploring biodegradative efficiency: a systematic review on the main microplastic-degrading bacteria

**DOI:** 10.3389/fmicb.2024.1360844

**Published:** 2024-03-18

**Authors:** Milena Roberta Freire da Silva, Karolayne Silva Souza, Fabricio Motteran, Lívia Caroline Alexandre de Araújo, Rishikesh Singh, Rahul Bhadouria, Maria Betânia Melo de Oliveira

**Affiliations:** ^1^Molecular Biology Laboratory, Department of Biochemistry, Federal University of Pernambuco - UFPE, Recife, PE, Brazil; ^2^Department of Civil and Environmental Engineering, Federal University of Pernambuco - UFPE, Recife, PE, Brazil; ^3^Amity School of Earth & Environmental Sciences, Amity University Punjab (AUP), Mohali, India; ^4^Department of Environmental Studies, Delhi College of Arts and Commerce, University of Delhi, New Delhi, India

**Keywords:** bacteria, bioremediation, synthetic polymers, microorganisms, xenobiotic

## Abstract

**Introduction:**

Microplastics (MPs) are widely distributed in the environment, causing damage to biota and human health. Due to their physicochemical characteristics, they become resistant particles to environmental degradation, leading to their accumulation in large quantities in the terrestrial ecosystem. Thus, there is an urgent need for measures to mitigate such pollution, with biological degradation being a viable alternative, where bacteria play a crucial role, demonstrating high efficiency in degrading various types of MPs. Therefore, the study aimed to identify bacteria with the potential for MP biodegradation and the enzymes produced during the process.

**Methods:**

The methodology used followed the Preferred Reporting Items for Systematic Reviews and Meta-Analyses (PRISMA) protocol.

**Results and Discussion:**

The research yielded 68 eligible studies, highlighting bacteria from the genera *Bacillus*, *Pseudomonas*, *Stenotrophomonas*, and *Rhodococcus* as the main organisms involved in MP biodegradation. Additionally, enzymes such as hydrolases and alkane hydroxylases were emphasized for their involvement in this process. Thus, the potential of bacterial biodegradation is emphasized as a promising pathway to mitigate the environmental impact of MPs, highlighting the relevance of identifying bacteria with biotechnological potential for large-scale applications in reducing MP pollution.

## 1 Introduction

The microplastics (MPs) are considered emerging contaminants due to their occurrence in different environmental compartments, including atmospheric, aquatic, and terrestrial. They are defined as plastic particles ranging in size from 1 μm to 5 mm and are found in various types, sizes, shapes, and primary and secondary polymeric compositions (Miri et al., 2022; Thakur et al., 2023).

Microplastics (MPs) are considered harmful to wildlife and humans due to their persistent properties and bioaccumulation. This is attributed to the addition of various substances during their manufacturing process, such as pigments, plasticizers, and flame retardants. Additionally, due to their chemical-physical characteristics, they exhibit high durability, requiring an extended period for degradation in the environment (Cai et al., 2023; Niu et al., 2023).

Therefore, the production of plastics in the industry has been going on since the 1950s, with annual production reaching around 2 million tons, so that in 2015 this production rose significantly to 380 million tons per year. As a result, looking back from 1950 to 2015, approximately more than 7,800 million tons of plastics were produced, resulting in approximately 6,300 million tons of waste. Over the past 70 years, global plastic production has increased from 1.5 million tons to approximately 359.0 million tons, with an estimated projection of reaching 500.0 million tons by 2025. This trend raises significant concerns within civil society, as MPs are primarily generated through the degradation of larger polymers, a process influenced by physical, chemical, or biological factors (Cverenkárová et al., 2021; Torena et al., 2021; Villalobos et al., 2022; Osman et al., 2023). As microplastics increasingly contaminate the environment, the food chain has also been significantly impacted. Plastic contamination has occurred in invertebrates such as polychaetes, 51 crustaceans, echinoderms, bivalves, and vertebrates, including fish, seabirds, and mammals. These particles have entered the food chain either directly or through trophic transfer. Indeed, one of the main concerns arising from microplastic contamination is its bioaccumulative effect in the digestive tract (Cverenkárová et al., 2021).

Microplastics (MPs) enter the environment through various pathways due to poor management and dumping practices. However, there are mechanisms that can be employed to control their presence in the environment, such as biological, thermal, and photocatalytic degradation. Biological degradation occurs through the use of different types of microorganisms, as some have the potential to be employed in bioremediation processes (Park and Kim, 2019).

These microorganisms are widely distributed in nature, with abundance among bacteria due to their rapid reproduction, diverse nutritional capabilities, strong adaptability, and significant potential for degrading MPs. They demonstrate high efficiency in degrading MPs such as Polyethylene terephthalate (PET), Polyethylene (PE), and Polypropylene (PP) in the natural environment (Yuan et al., 2020; Li et al., 2022). Although polymers have a relatively simple chemical structure, they are known for their high resistance to biodegradation, especially due to their hydrophobic structure, high molecular weight, and lack of a favorable functional group. Consequently, when present in the environment in combination with biotic and abiotic factors, they can undergo transformations leading to the formation of alcoholic or carbonyl groups. This process increases plastic hydrophilicity and provides anchors that facilitate the attachment of bacterial species (Villalobos et al., 2022; Pathak, 2023; Thakur et al., 2023).

Thus, exploring the capability of bacteria and the interaction between bacterial enzymes and microplastics is crucial for obtaining and identifying key microorganisms with potential for bioremediation through the biodegradation of synthetic polymers. Therefore, the present study aims to identify the main bacteria that demonstrate viability for the biodegradation of MPs in various environments, as well as the enzymes produced during the degradation process.

## 2 Materials and methods

### 2.1 Protocol

This systematic review was conducted in accordance with the Preferred Reporting Items for Systematic Reviews and Meta-Analyses (PRISMA) protocol, organized into the respective phases of planning, execution, and data reporting.

### 2.2 Eligibility criteria

For the conduct of this investigation, the PECO strategy was employed: Population—Microorganisms, Exposure—Microplastics, Comparison—Not applicable, and Outcomes—Potential of bacteria for microplastic biodegradation.

Thus, according to the aforementioned strategy, studies that considered the key microorganisms involved in microplastic biodegradation were deemed eligible without restrictions on the year and/or language. Consequently, the exclusion criteria encompassed studies and editorial files, typical discussion documents, comments, letters, reviews, studies with incomplete or insufficient data regarding methodology and microorganism identification, as well as duplicates and titles that did not align with the proposed theme.

### 2.3 Information and research sources

Searches were conducted in the electronic databases PubMed, Medline, and LILACS. Subsequently, the definition of Medical Subject Headings (MeSH) and Health Sciences Descriptors (Decs) descriptors and synonyms, in addition to keywords and Boolean operators, was carried out for the composition of the controlled search strategy. Thus, the terms “Microplastics” AND “Bacteria” AND “Ecosystem” AND “Environment” AND “Biodegradation” AND “Bioremediation” were obtained.

### 2.4 Articles selection

For study selection, two reviewers participated independently and blindly, resulting in the following stages for the inclusion and exclusion of studies. The first stage involved title analysis, excluding duplicates. The second stage involved discussing eligibility criteria separately according to the PECO strategy, enabling the exclusion of studies not related to the proposed strategy. The third stage consisted of eliminating studies after reading the abstracts, which could not provide sufficient information and data for the fulfillment of the current proposal.

### 2.5 Data collection process

Subsequently, following the selection of studies, information from the main data of eligible studies was extracted using a form created by the authors with predefined items. The key items included: first author, year of publication, genus and species of the isolated microorganism, source of microorganisms, type of microplastics, and microbial enzymes with potential for microplastic biodegradation. The aforementioned data was then tabulated in an Excel spreadsheet, and any additional calculations and necessary tabulations were performed by two researchers.

### 2.6 Bias risk

The publication bias was assessed using the Joanna Briggs Institute’s (JBI) critical appraisal checklist for qualitative research (Lockwood et al., 2017). This checklist involves three respective classifications: High, Moderate, and Low. A High-risk rating results in more than 49% scoring “yes,” Moderate involves achieving 50–69% scoring “yes,” and Low consists of a score of “yes” ≥ 70%. According to this assessment, studies with a high risk of publication bias will be excluded.

## 3 Results

**FIGURE 1:**
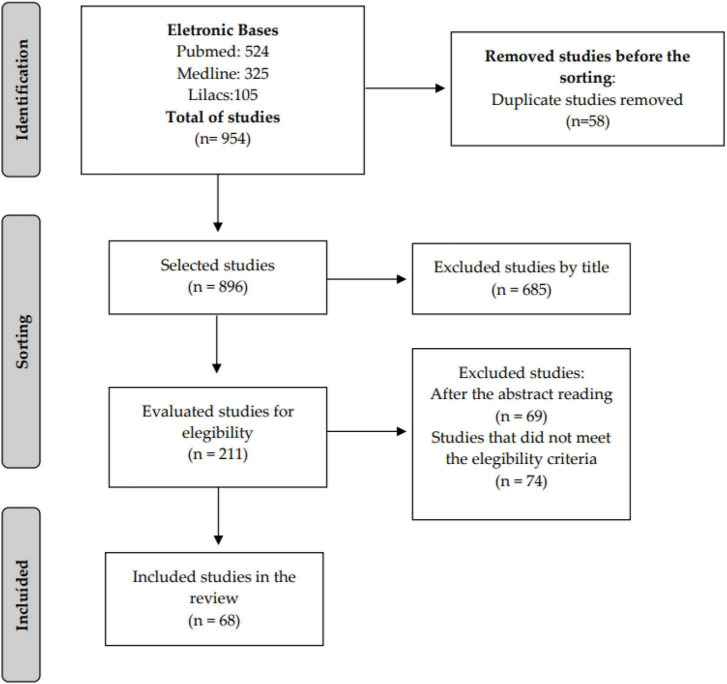
Flowchart with quantitative and qualitative data of excluded and included articles.

In this systematic search, initially, 954 studies were found. Of these, 58 were excluded due to duplication, 685 due to title, 69 due to abstract, and 74 did not meet eligibility criteria, resulting in a total of 68 eligible studies for systematic review. Figure 1 presents the flowchart demonstrating the main quantitative and qualitative data of the excluded and included articles.

According to the eligible studies, the first analysis was conducted to identify the pre-dominance of microorganisms with the potential for microplastic biodegradation. Table 1 presents the main genera and species of microorganisms and their action in the biodegradation of different types of microplastics and the main enzymes analysis related to the degradation of microplastics.

**TABLE 1:** Qualitative synthesis of the main genera and species of microorganisms with potential for microplastic biodegradation.

Genus	Species	Source	Microplastic	Enzymes	Analysis of biodegradation	References
*Achromobacter*	*Achromobacter xylosoxidans*	Garbage disposal	HDPE, PCL	Lipase	ATR-FTIR	Oda et al., 1997; Kowalczyk et al., 2016
*Actinomycetes*	*Actinomycetes* sp.	Mangroves	PP	–	Determination of dry weight	Auta et al., 2017
*Alcaligenes*	*Alcaligenes faecalis*	Laboratory isolate	PCL	–	HPLC	Oda et al., 1997
*Alcanivorax*	*Alcanivorax borkumensis*	Marine environment	LDPE	–	ATR-FTIR	Delacuvellerie et al., 2019
*Alicycliphilus*	*Alicycliphilus* sp.	Garbage disposal	PU	Esterase	IRS-FTIR, SEM, HPLC	Oceguera-Cervantes et al., 2007
*Aneurinibacillus*	*Aneurinibacillus* sp.	Waste management landfills and sewage treatment plants, mangroves	HDPE, LDPE, PP	–	Determination of dry weight, AFM, EDS, NMR, FTIR, SEM	Auta et al., 2017; Skariyachan et al., 2018
*Arthrobacter*	*Arthrobacter* sp.	Marine environment	HDPE	–	FTIR	Balasubramanian et al., 2010
*Azotobacter*	*Azotobacter vinelandii*	Laboratory isolate	PHB	PHB-depolymerase	HPLC	Adaya et al., 2018
*Bacillus*	*Bacillus* sp.	Landfill, mangrove sediment	PE, PP	Oxidoreductase, alkane- monooxygenase, hydrolases	Determination of dry weight	Auta et al., 2018; Wei et al., 2018; Park et al., 2019
	*Bacillus cereus*	Waste disposal, landfill, mangroves, marine environment	HDPE, PET, PP	Hydrolase, Oxidoreductase, laccase, and alkane hydroxylase	Determination of dry weight, FTIR	Satlewal et al., 2008; Sudhakar et al., 2008; Auta et al., 2017, 2018; Muhonja et al., 2018; Zerhouni et al., 2018; Maroof et al., 2021
	*Bacillus vallismortis*	Cow dung	HDPE	Hydrolase and oxidoreductase	AFM, EDS, NMR, FTIR, SEM	Skariyachan et al., 2018
	*Bacillus siamensis*	Garbage disposal	LDPE	Laccase and alkane hydroxylase	FTIR, X-ray diffraction (XRD)	Maroof et al., 2021
	*Bacillus wiedmannii*	Garbage disposal	LDPE	Laccase and alkane hydroxylase	FTIR, XRD	Maroof et al., 2021
	*Bacillus subtilis*	Garbage disposal, marine environment, soil	LDPE, PE, PS, PUR	Laccase and alkane hydroxylase, esterase	MEV, FTIR	Harshvardhan and Jha, 2013; Shah et al., 2013; Asmita et al., 2015; Maroof et al., 2021
	*Bacillus niacini*	Activated sludge in a wastewater treatment plant	PVA	PVAase	Determination of dry weight	Bian et al., 2019
	*B. paralicheniformis*	Marine deep-sea sediment	PS	Peroxidase, esterase, dioxygenase and monooxygenase	TG-DSC, SEM, NMR, FTIR	Kumar et al., 2021
	*Bacillus gottheilii*	Mangroves	PE, PET, PP, PS	–	Determination of dry weight	Auta et al., 2017
	*Bacillus brevies*	Soil	PE	–	MEV	Kuroki et al., 2009
	*Bacillus pumilus*	Laboratory insulation, garbage dump, soil	PE, LDPE	–	MEV, FTIR, GC-MS	Roy et al., 2008; Satlewal et al., 2008; Nowak et al., 2011; Harshvardhan and Jha, 2013
	*Bacillus sphericus*	Marine environment	LDPE	–	FTIR	Sudhakar et al., 2008
	*Bacillus amyloliquefaciens*	Garbage disposal	LDPE	–	Determination of dry weight	Das and Kumar, 2013
*Brevibacillus*	*Brevibacillus* sp.	Waste management landfills and sewage treatment plants	PE, PP	–	AFM, EDS, NMR, FTIR, SEM	Skariyachan et al., 2018
	*Brevibacillus borstelensis*	Soil	PE	–	FTIR	Hadad et al., 2005
*Citrobacter*	*Citrobacter* sp.	Intestinal isolates in larvae of *Tenebrio molitor*	PS	–	FTIR, NMR	Brandon et al., 2018
*Cryptococcus*	*Cryptococcus* sp.	Laboratory isolate	PLA	Cutinase	Determination of dry weight	Masaki et al., 2005
*Cupriavidus*	*Cupriavidus* sp.	Marine litter and water	PVC	–	TGA, GPC	Giacomucci et al., 2020
	*Cupriavidus necator*	Laboratory isolate	LDPE	–	FTIR	Montazer et al., 2019
*Desulfovibrio*	*Desulfovibrio* sp.	Marine litter and water	PVC	–	TGA, GPC	Giacomucci et al., 2020
*Exiguobacterium*	*Exiguobacterium* sp.	Soil	PS	Oxygenase	FTIR	Parthasarathy et al., 2022
*Ideonella*	*Ideonella sakaiensis*	Laboratory isolate, sediments, soil, wastewater and activated sludge	PET	PETase, MHETase, glycosidic hydrolases	FTIR	Tanasupawat et al., 2016; Liu et al., 2019; Palm et al., 2019
*Klebsiella*	*Klebsiella pneumoniae*	Laboratory isolate	HDPE	Lipase	AFM, UTM, FTIR, SEM	Awasthi et al., 2017
*Lysinibacillus*	*Lysinibacillus* sp.	Soil, laboratory isolate	PE, PP	–	GC-MS, FTIR, SEM, XRD	Esmaeili et al., 2013; Mukherjee et al., 2016; Jeon et al., 2021
	*Lysinibacillus xylanilyticus*	Landfill	LDPE	–	FTIR, SEM, XRD	Esmaeili et al., 2013
*Microbacterium*	*Microbacterium paraoxydans*	Laboratory isolate	LDPE	–	ATR-FTIR	Rajandas et al., 2012
*Micrococcus*	*Micrococcus luteus*	Laboratory isolate	LDPE	–	FTIR	Montazer et al., 2019
*Mycobacterium*	*Mycobacterium neoaurum*	Soil	Dimethylphenol	–	HPLC	Xiong et al., 2020
*Oscillatoria*	*Oscillatoria subbrevis*	Domestic sewage water	PE	–	Determination of dry weight	Sarmah and Rout, 2018
*Paenibacillus*	*Paenibacillus* sp.	Landfill	PE	Alkane monooxygenase	FTIR, SEM	Bardají et al., 2019; Park et al., 2019
*Pseudomonas*	*Pseudomonas* sp.	Soil, Antarctic soil	BPA, PP, PE, PET, PS	Alkane hydroxylase	HPLC, FTIR	Matsumura et al., 2009; Jeon and Kim, 2015; Wilkes and Aristilde, 2017; Habib et al., 2020; Taghavi et al., 2021
	*Pseudomonas fluorescens*	Soil, garbage disposal	PE	Alkane hydroxylase	FTIR	Balasubramanian et al., 2010; Nowak et al., 2011; Jeon and Kim, 2015; Thomas et al., 2015
	*Pseudomonas aeruginosa*	Garbage dump, laboratory insulation, surface water, soil	LDPE, PE, PLA, PS	Alkane hydroxylase	ATR-FTIR	Rajandas et al., 2012; Shimpi et al., 2012; Yoon et al., 2012; Tribedi and Sil, 2013; Taghavi et al., 2021; Tamnou et al., 2021
	*Pseudomonas aestusnigri*	Marine environment	PU	Polyester hydrolase	IMAC, SEC	Bollinger et al., 2020
	*Pseudomonas protegens*	Laboratory isolate	PU	Lipase	NMR, HPLC	Hung et al., 2016
	*Pseudomonas geniculata*	Soil and wastewater sludge	PLA	Protease	GPC, FTIR	Bubpachat et al., 2018
	*Pseudomonas citronellolis*	Landfill	LDPE	–	SEM, FTIR	Bhatia et al., 2014
*Pseudozyma*	*Pseudozyma antártica*	Soil	Biodegradable plastic	Esterase	SEM	Sameshima-Yamashita et al., 2019
*Rhodococcus*	*Rhodococcus* sp.	Antarctic soil, mangrove sediment, laboratory isolate	PP	Monooxygenase, hydrolases	Determination of dry weight, FTIR	Auta et al., 2018; Habib et al., 2020
	*Rhodococcus ruber*	Laboratory isolate	PE, PS	Laccase, hydrolases	Determination of dry weight, GPC	Mor and Sivan, 2008; Santo et al., 2013
	*Rhodococcus rhodochrous*	Laboratory isolate	PE	–	FTIR	Fontanella et al., 2010
*Serratia*	*Serratia* sp.	Intestinal isolates in larvae of *Galleria mellonella* L.	PS	–	FTIR	Lou et al., 2022
*Sporobacter*	*Sporobacter* sp.	Marine litter and water	PVC	–	TGA, GPC	Giacomucci et al., 2020
*Sporosarcina*	*Sporosarcina globispora*	Mangroves	PP	–	Determination of dry weight	Auta et al., 2017
*Staphylococcus*	*Staphylococcus* sp.	Garbage disposal	PP	–	FTIR, SEM	Oliya et al., 2020
	*Staphylococcus aureus*	Soil	PS	–	MEV, FTIR	Asmita et al., 2015
*Stenotrophomonas*	*Stenotrophomonas* sp.	Soil	Nylon	–	SEM, MALDI-TOF	Tachibana et al., 2010
	*Stenotrophomonas rhizophila*	Forest	PVA	PVA-dehydrogenase	MAF	Wei et al., 2019
	*Stenotrophomonas panacihumi*	Garbage disposal	PP	–	Determination of dry weight	Jeon et al., 2016
	*Stenotrophomonas pavanii*	Garbage disposal	LDPE	–	XRD, SEM	Mehmood et al., 2016
*Streptococcus*	*Streptococcus pyogenes*	Soil	PS	–	MEV, FTIR	Asmita et al., 2015
*Streptomyces*	*Streptomyces* sp.	Marine environment	PET, PCL	Lacase, SM14est (PETase)	GC-MS, FTIR, NMR	Alshehrei, 2017; Almeida et al., 2019
	*Streptomyces bangladeshensis*	Soil	PHB	PHB depolymerase	FTIR	Hsu et al., 2012
*Vibrio*	*Vibrio* sp.	Solid waste dumped into water bodies	PET	–	FTIR, SEM, XRD	Sarkhel et al., 2020

The literature describes different types of microplastics (MPs), and for this study, the biodegradation actions were investigated for the following types: Polyethylene (PE), Poly-propylene (PP), Polyvinyl chloride (PVC), Polyethylene terephthalate (PET), Polystyrene (PS), High-density polyethylene (HDPE), Low-density polyethylene (LDPE), Polycaprolactone (PCL), Polyhydroxybutyrate (PHB), Polylactic acid (PLA), Bisphenol (Dimethylphenol, BPA), Polyurethane (PU), Biodegradable plastic, Nylon, and Polyvinyl acetate (PVA).

Also for this study, biodegradation analysis actions were investigated: attenuated total reflectance Fourier transform infrared spectroscopy (ATR-FTIR) as well as electron microscope (SEM), Fourier transform infrared spectroscopy (IRS-FTIR), and gas chromatography-mass spectrometry analyses of hydroform (GC-MS), high-performance liquid chromatography (HPLC), atomic force microscopy (AFM), energy dispersive spectroscopy (EDS), nuclear magnetic resonance (NMR), X-ray diffraction (XRD), Fourier transform infrared spectroscopy (FTIR), scanning electron microscopy (SEM), thermogravimetry and differential scanning calorimetry (TG-DSC), nuclear magnetic resonance (NMR), gel permeation chromatography (GPC), thermogravimetric analysis (TGA), universal tensile machine (UTM), atomic force microscope (AFM), immobilized metal ion affinity chromatography (IMAC), size exclusion chromatography (SEC), gel permeation chromatography (GPC), matrix assisted laser desorption/ionization time of flight (MALDI TOF), mobile amorphous fraction (MAF) and determination of dry weight.

A total of 34 different bacterial genera and 63 species were observed. The most frequently found genera were *Bacillus* (*n* = 20), *Pseudomonas* (*n* = 14), *Stenotrophomonas* (*n* = 4), *Rhodococcus* (*n* = 3), and their respective species. For the genus *Bacillus*, the following species were identified: *Bacillus* sp., *B. cereus*, *B. vallismortis*, *B. siamensis*, *B. wiedmannii*, *B. subtilis*, *B. niacini*, *B. paralicheniformis*, *B. gottheilii*, *B. brevies*, *B. pumilus*, *B. sphericus*, and *B. amyloliquefaciens.* For the genus *Pseudomonas*, the identified species included *Pseudomonas* sp., *P. fluorescens*, *P. aeruginosa*, *P. aestusnigri*, *P. protegens*, *P. geniculata*, and *P. citronellolis*. *Stenotrophomonas* genus included the species *Stenotrophomonas* sp., *S. rhizophila, S. panacihumi*, and *S. pavanii*. For the genus *Rhodococcus*, the identified species were *Rhodococcus* sp., *R. ruber*, and *R. rhodochrous*.

In the qualitative synthesis regarding the main enzymes found with biodegradation activities, hydrolases and alkane hydroxylase were described as more abundant, especially for the genera *Bacillus*, *Pseudomonas*, *Rhodococcus*, and *Ideonella*.

Regarding the bias risk from the JBI checklist, it was observed that the majority of responses to the critical appraisal questionnaire from the 68 studies consisted of > 80% “Yes” responses. This indicates that the eligible studies in this investigation had a low risk of bias, meaning they demonstrated high methodological quality.

## 4 Discussion

The degradation of MPs in the environment is considered an integrated process, involving biological, physical, and chemical actions. Studies have shown that biodegradation has been the most frequent and represents a future perspective for reducing these pollutants in aquatic and terrestrial environments, known as bioremediation (Yuan et al., 2020).

Thus, this work aimed to conduct a literature review on the main bacteria and microbial enzymes involved in the degradation of MPs.

Approximately 80% of commercially marketed plastic materials are obtained from thermoplastic polymers, named for their ability to change from a solid to a viscous state when subjected to high temperatures. The main industrial polymers derived from these thermoplastics and marketed worldwide are Polyethylene, Polypropylene, Polyvinyl chloride, Polyethylene terephthalate, and Polystyrene (Kotova et al., 2021).

This review found that the degradation of MPs through microbial biodegradation can occur in various sediments, including wastewater, landfill deposits, sanitary landfills, sewage residues, soil, among others (Yuan et al., 2020). This occurs because MPs represent a favorable compartment for bacterial colonization and growth, mainly by providing carbon as an energy source (Rujnić-Sokele and Pilipović, 2017). Therefore, studying pure cultures of bacterial isolates is advantageous most of the time, as it enables a controlled analysis of the metabolic pathways of these respective MPs degrading organisms. In this study, the main species and bacterial enzymes (Table 1) involved in this process of MP degradation can be observed, although this data is still not sufficient to understand the entire degradation mechanism (Bacha et al., 2021).

Over the years, an increase in the number of bacterial species with the potential for MP degradation has been observed. The most reported genera are *Bacillus*, *Pseudomonas*, *Stenotrophomonas*, and *Rhodococcus* (Auta et al., 2018; Wei et al., 2018; Amobonye et al., 2021; Li et al., 2023; Thakur et al., 2023).

The action of these bacteria occurs mainly by forming pores and irregularities on the surfaces of MPs, making them rough with various grooves and fissures, as well as by gaining the ability to adhere, colonize, and damage the MPs (Du et al., 2021; Golmohammadi et al., 2023).

Auta et al. (2017, 2018), used isolates from the genera *Rhodococcus* sp. and *Bacillus* sp. and detected a weight reduction of PP by 6.4 and 4.0%, respectively, after a period of just over a month of incubation with the MPs. Additionally, the authors found that the species *B. cereus* and *B. gottheilii* showed degradative capacity for PE of 1.6 and 6.2%, for PET of 6.6 and 3.0%, and for PS of 7.4 and 5.8%, respectively. In this perspective, Shimpi et al. (2012) identified a biodegradative capacity of 10% for PS and PLA by the species *P. aeruginosa.*

The studies demonstrate that these microorganisms not only cause changes in the appearance of MPs but also enable conformational changes in their structures, especially in the functional groups, in addition to reducing the molecular weight and tensile properties, as seen in the work of Yuan et al. (2020) using *Stenotrophomonas maltophilia*.

Biodegradation of some plastic materials such as PVC and PET is challenging because PVC contains various additives in its composition, such as plasticizers, heat stabilizers, flame retardants, and/or biocides, resulting in a total weight of approximately 50–75% of the final material. PET, due to its high content of aromatic terephthalate elements, limits the mobility of the polymeric chains, making it highly resistant to degradation by bacteria (Kotova et al., 2021).

Thus, it can be observed that the respective studies addressed have shown a more significant effect on the degradation of modified plastics such as PS, PE, and PLA, which can be explained by these plastic materials presenting better biodegradability (Chandra and Singh, 2020; Yuan et al., 2020).

Initially, the biodegradation of MPs by bacteria occurs from the degradation of larger polymer structures to smaller particles, consequently, degradation into oligomers, dimers, and monomers, finally leading to mineralization through microbial biomass. Therefore, this decomposition is aided by a diversity of enzymes that produce intermediate products (Miri et al., 2022).

Such bacterial enzymes with the potential for biodegradation are demonstrated in the studies of Shahnawaz et al. (2019), Taniguchi et al. (2019), and Sol et al. (2020). These studies reinforce that extracellular enzymes are the most studied in the literature, such as esterases, lipases, lignin peroxidases, laccases, depolymerases, cutinases, and manganese peroxidases, as they increase the hydrophilicity of MPs, allowing the conversion of carboxylic and/or alcoholic groups and significantly improving bacterial attachment and the degradation of these compounds.

Thus, the biodegradation of microplastics by bacteria through enzymes is capable of digesting these particles into carbon sources, thus changing the structure, function, molecular weight, etc., making it less toxic to the environment. Therefore, the main products obtained after mineralization by biodegradation of microplastics by bacteria are CO_2_ and H_2_O molecules (Anand et al., 2023).

The biodegradation of MPs by bacteria has been significantly reported in several studies as a bioremediation factor for the elimination of these compounds in the environment, as plastic materials have been increasingly used extensively and indiscriminately, causing pollution in terrestrial and aquatic environments and even impacting the public and health due to its cumulative effect. Thus, promising biotechnological techniques such as biodegradation of MPs by bacteria, however, is a challenging approach, given its high cost, since the species of bacteria and their main enzymes involved in the degradation process is still considered a high-quality treatment. Therefore, studies have intensified so that this biotechnological tactic can be incorporated into practice in order to reduce its cost, be reproducible and apply it appropriately on a large scale. Therefore, even though it is a methodology with a future perspective, it is still necessary at present for there to be a worldwide economy of polymers so that it can be directed toward a green and sustainable environmental future.

## 5 Conclusion

The findings in this investigation highlighted that the genera *Bacillus*, *Pseudomonas*, *Stenotrophomonas*, and *Rhodococcus*, along with their corresponding species and enzymes—hydroxylases, lipases, proteases, esterases, hydrolases, and laccases—were the main ones reported in the scientific literature regarding the potential for MP biodegradation. This indicates that these microorganisms can act as functional agents in reducing MPs.

Therefore, studies like this emphasize the importance of conducting further research, especially considering the establishment of protocols with experiments under real environmental conditions. This is crucial so that, in the future, the interaction of bacteria with MPs holds practical and biotechnological value on a large scale, aiming to reduce the impacts caused by these compounds in the environment.

## Data availability statement

The datasets presented in this study can be found in online repositories. The names of the repository/repositories and accession number(s) can be found below: PubMed, Medline, and LILACS.

## Author contributions

MS: Writing – original draft, Writing – review & editing. KS: Formal Analysis, Investigation, Methodology, Validation, Writing – original draft, Writing – review & editing. FM: Writing – original draft, Writing – review & editing. LA: Writing – original draft, Writing – review & editing. RS: Writing – original draft, Writing – review & editing. RB: Writing – original draft, Writing – review & editing. MO: Supervision, Writing – original draft, Writing – review & editing.
